# Supplementary material for: Prenatal exposure to Zika virus shapes offspring neutrophil function in a sex-specific manner
Source: Nat Commun. 2025 Oct 3;16:8839. doi: 10.1038/s41467-025-63941-x (PMC12494944; doi:10.1038/s41467-025-63941-x)
Supplement: Supplementary file 1 — Supplementary Information [file 41467_2025_63941_MOESM1_ESM.pdf]

Title: Prenatal Exposure to Zika Virus Shapes Offspring Neutrophil Function in a Sex-Specific Manner

Sample ID	Tissue Type	PCR (Cq value)	Negative Control (Cq value)	Positive Control (Cq value)	Result Interpretation
SDE-Z1L2	Placenta	35.51	33.36	17.56	No virus
SDE-Z1L3	Placenta	35.92	33.36	17.56	No virus
SDE-Z1R1	Placenta	36.69	33.36	17.56	No virus
SDE-Z1R2	Placenta	35.28	33.36	17.56	No virus
SDE-Z2L1	Placenta	36.20	33.36	17.56	No virus
SDE-Z2L2	Placenta	35.51	33.36	17.56	No virus
SDE-Z2L5	Placenta	35.90	33.36	17.56	No virus
SDE-Z2R1	Placenta	35.89	33.36	17.56	No virus
SDE-Z3R2	Placenta	35.72	33.36	17.56	No virus
SDE-Z3R3	Placenta	35.78	33.36	17.56	No virus
SDL-Z1L1	Placenta	37.10	33.36	17.56	No virus
SDL-Z1L2	Placenta	35.32	33.36	17.56	No virus
SDL-Z1R1	Placenta	35.45	33.36	17.56	No virus

Supplemental Table 1. qRT-PCR analysis of placenta tissue for viral presence.

Table showing PCR cycle threshold (Cq) values from placenta tissues and corresponding negative and positive control. Cq values from all placenta samples were similar to the negative control (no virus), indicating no detectable viral titer in the tissues. Due to the large number of samples analyzed (n = 34), only a subset of representative data is shown here to illustrate the overall findings.

Groups	Well	Mo Eotaxin (74)	Mo G-CSF (54)	Mo GM-CSF (73)	Mo IFN-g (34)	Mo IL-1a (53)	Mo IL-1b (19)	Mo IL-2 (36)	Mo IL-3 (18)
Control	SDE C1	522.5	63.5	23	21.5	46.5	10	18	12
	SDE C2	630.84	173.95	123.07	36.09	27.17	39.3	61.44	12.46
	SDE C3	711.89	59.89	102.22	23.72	13.04	27.2	48.59	8.83
	C11	607.39	150.40	129.16	31.94	17.12	35.09	52.94	12.46
	C14	300.38	171.35	116.8	26.18	12.12	27.2	48.59	9.73
	C15	288.71	186.84	114.23	25.36	12.12	29.64	47.49	9.73
ZIKV	SDE Z1	584	67	24	23	17	11	16.5	14
	SDE Z2	773.53	100.27	124.3	48.61	33.04	34.49	59.34	19.4
	SDE Z3	563.78	232.39	129.16	29.06	16.55	29.64	48.59	10.64
	Z8	290.96	167.45	114.23	23.72	13.64	30.25	46.39	9.28
	Z9	505.29	139.78	124.3	27	12.43	32.07	57.22	11.09
	Z11	481.51	153.04	119.33	27	11.19	27.2	61.44	9.28
Groups	Well	Mo IL-4 (39)	Mo IL-5 (52)	Mo IL-6 (38)	Mo IL-9 (33)	Mo IL-10 (56)	Mo IL-12(p40) (76)	Mo IL-12(p70) (78)	Mo IL-13 (37)
Control	SDE C1	14	13	23.5	19	21	464.5	38	14
	SDE C2	15.45	21.84	25.18	78	267.29	571.68	152.5	262.25
	SDE C3	8.35	20.01	18.99	55.46	139.08	532.83	84.61	181.71
	C11	11.43	31.81	24.21	65.15	218.98	567.17	119.49	200.14
	C14	7.91	22.75	20.54	56.69	152.17	499.04	77.7	200.14
	C15	8.78	31.81	21.7	51.72	170.82	710.17	74.25	209.22
ZIKV	SDE Z1	15	12	19	22	23	458	44	15
	SDE Z2	17.93	25.48	23.24	89.34	232.16	934.36	239.39	236.03
	SDE Z3	8.78	24.57	23.63	62.15	188.5	690.88	96.18	209.22
	Z8	7.91	20.01	21.31	55.46	152.17	661.42	65.08	200.14
	Z9	8.78	23.66	22.86	59.13	185.61	576.19	84.61	222.71
	Z11	8.35	30.01	21.7	55.46	139.08	555.35	89.23	218.23

Groups	Well	Mo IL-17A (72)	Mo KC (57)	Mo MCP-1 (51)	Mo MIP-1a (77)	Mo MIP-1b (75)	Mo RANTES (55)	Mo TNF-a (21)	
Control	SDE C1	97	36	19	16	38	51	23	
	SDE C2	120.46	80.97	786.05	7.89	330.77	138.49	261.96	
	SDE C3	26.97	49.81	606.76	7.14	258.2	93.36	184.49	
	C11	68.92	72.09	801.99	9.14	363.03	84.69	254.14	
	C14	22.58	65.65	646.22	7.64	293.38	73.95	199.84	
	C15	20.59	63.05	736.42	7.14	268.53	75.77	199.84	
ZIKV	SDE Z1	145	42	15	18	46	36	25	
	SDE Z2	119.64	76.55	736.42	8.64	321.66	186.22	325.1	
	SDE Z3	49.68	61.75	701.6	7.64	330.77	96.76	199.84	
	Z8	17.01	61.75	636.57	7.39	270.23	70.27	184.49	
	Z9	38.59	66.95	701.6	8.39	294.99	77.58	230.76	
	Z11	47.86	65.65	616.85	6.89	283.59	79.37	199.84	

Supplemental Table 2. Maternal serum cytokine profiling at E12.5 following Zika virus infection.

Maternal serum (*n* = 6) was collected at embryonic day 12.5 (E12.5) from control and ZIKV-exposed pregnant mice. A 23-plex cytokine panel assay was performed to assess systemic inflammatory responses. No significant differences in cytokine levels (pg/mL) were observed between control and Zika-infected groups, indicating that maternal systemic inflammation was not significant at E12.5 (4 days post infection). Two-sided Student’s t-test was used.

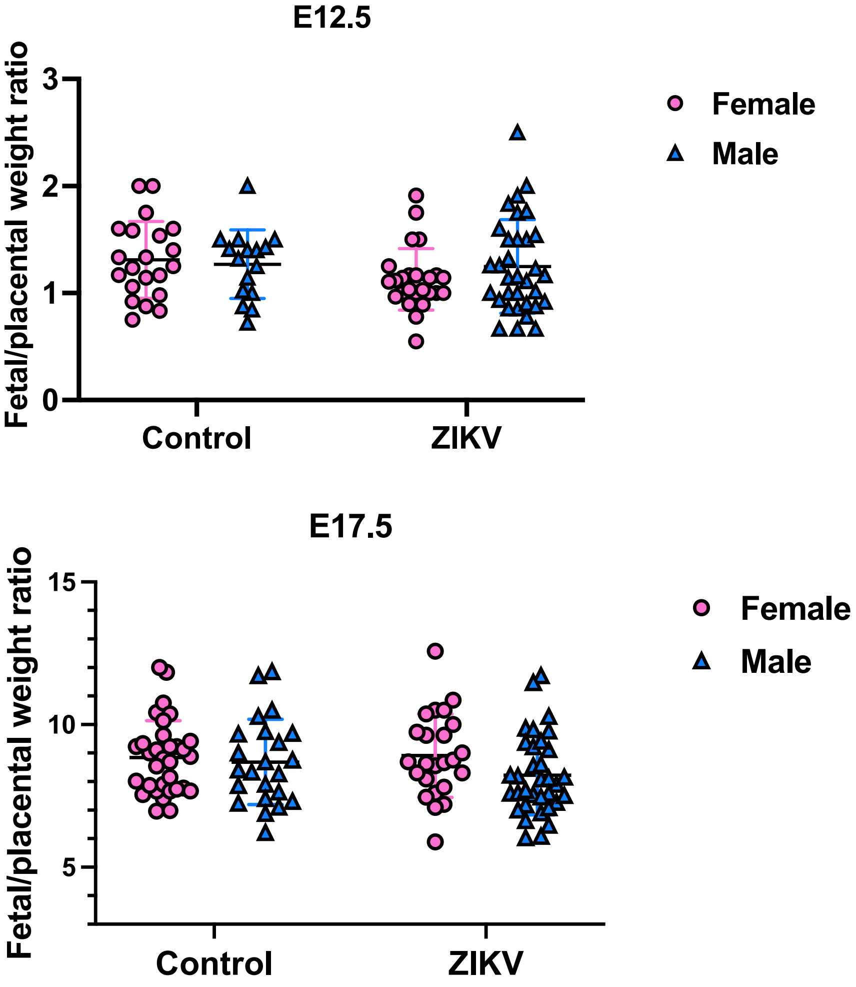

Supplemental Figure 1. Placental efficiency, calculated as the fetal-to-placenta weight ratio, at E12.5 and E17.5 in control and ZIKV-exposed pregnancies. *n* = 21 female, 16 male placentas/fetuses from 5 control dams; *n* = 25 female, 33 male placentas/fetuses from 7 ZIKV dams at E12.5 by sex. *n* = 32 female, 23 male placentas/fetuses from 7 control dams; *n* = 24 female, 34 male placentas/fetuses from 7 ZIKV dams at E17.5 by sex. Values represent mean ± SD. Statistical significance determined by two-way ANOVA with Šídák's multiple comparisons test.

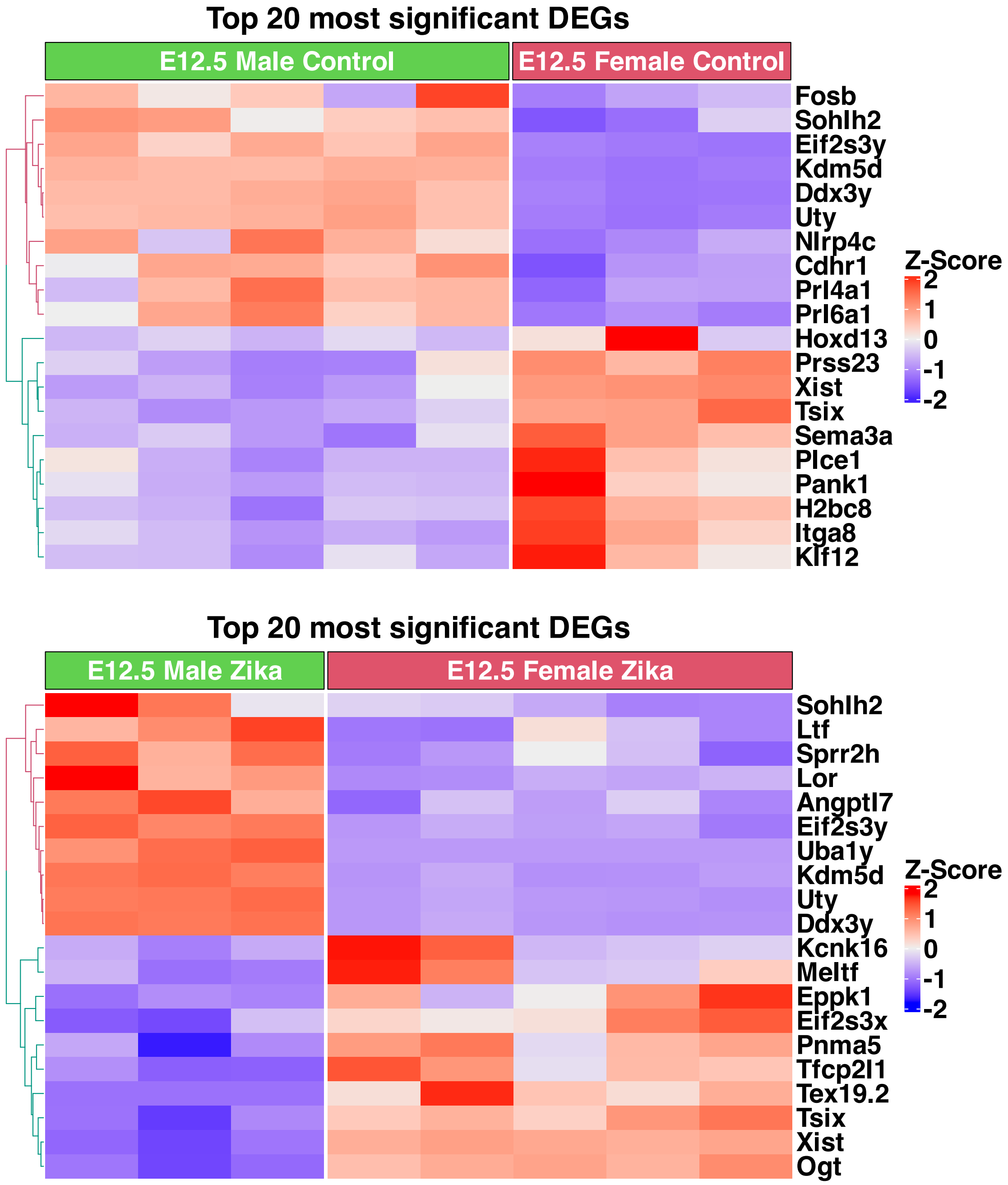

Supplemental Figure 2. Heatmaps showing the top 20 differentially expressed genes (DEGs) (adjusted p value < 0.05) between E12.5 female and male placentas in control and ZIKV-exposed groups. These comparisons illustrate baseline sex-dimorphic gene expression differences and sex-specific transcriptional responses to prenatal ZIKV exposure.

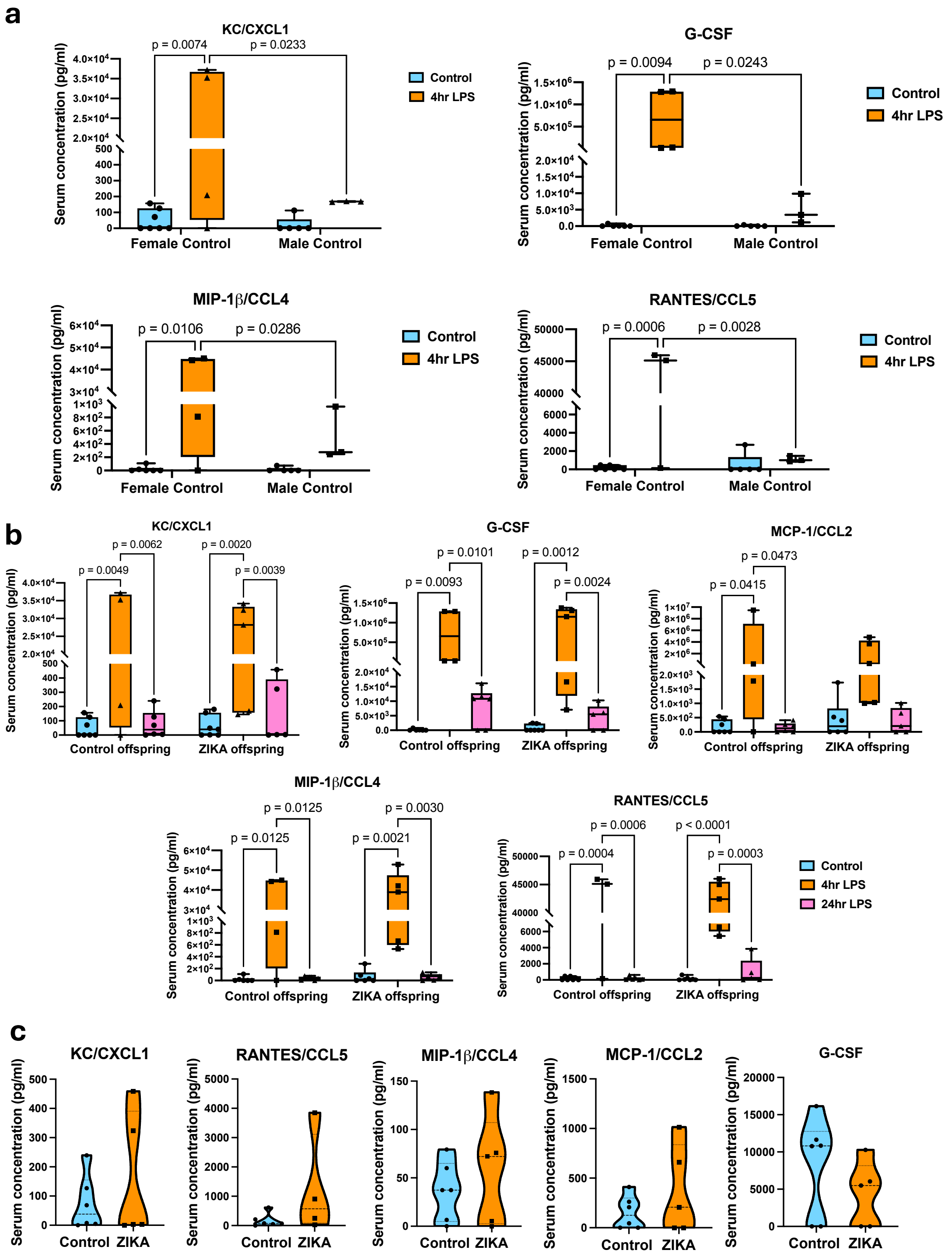

a
KC/CXCL1
G-CSF
MIP-1β/CCL4
RANTES/CCL5
Control
4hr LPS
Female Control
Male Control
Serum concentration (pg/ml)
p = 0.0074
p = 0.0233
p = 0.0094
p = 0.0243
p = 0.0106
p = 0.0286
p = 0.0006
p = 0.0028
b
KC/CXCL1
G-CSF
MCP-1/CCL2
MIP-1β/CCL4
RANTES/CCL5
Control offspring
ZIKA offspring
p = 0.0049
p = 0.0062
p = 0.0020
p = 0.0039
p = 0.0093
p = 0.0101
p = 0.0012
p = 0.0024
p = 0.0415
p = 0.0473
p = 0.0125
p = 0.0125
p = 0.0021
p = 0.0030
p = 0.0004
p = 0.0006
p < 0.0001
p = 0.0003
Control
4hr LPS
24hr LPS
c
KC/CXCL1
RANTES/CCL5
MIP-1β/CCL4
MCP-1/CCL2
G-CSF
Control
ZIKA

Supplemental Figure 3. Effect of maternal Zika infection on female offspring cytokine response to LPS.

Male and female offspring from control and Zika-exposed groups were treated with lipopolysaccharide (LPS) (0.8 mg/kg) intraperitoneally at PND35. (a) Serum concentrations of CXCL1, G-CSF, MIP-1β and RANTES were quantified 4 hours post-LPS injection in control offspring to compare baseline inflammatory responses between sexes. *n* = 7 for female control, 4 for female 4 h LPS, 5 for male control and 3 for male 4 h LPS. (b) Serum levels of CXCL1, G-CSF, MCP-1, MIP-1β, and RANTES at 4 and 24 hours post-LPS treatment in female offspring. *n* = 7 for control untreated, 4 for control 4 h LPS, 6 for control 24 h LPS; *n* = 7 for ZIKV untreated, 5 for ZIKV 4 h LPS, 5 for ZIKV 24 h LPS. (c) Comparative analysis of serum levels of CXCL1, G-CSF, MCP-1 , MIP-1β and RANTES at 24 hours post-LPS treatment in female offspring. *n* = 6 for control and 5 for ZIKV. Data are shown as the mean ± SD. Two-way ANOVA with Šídák's multiple comparisons (a, b) and two-sided Student’s t-test (c) were used.

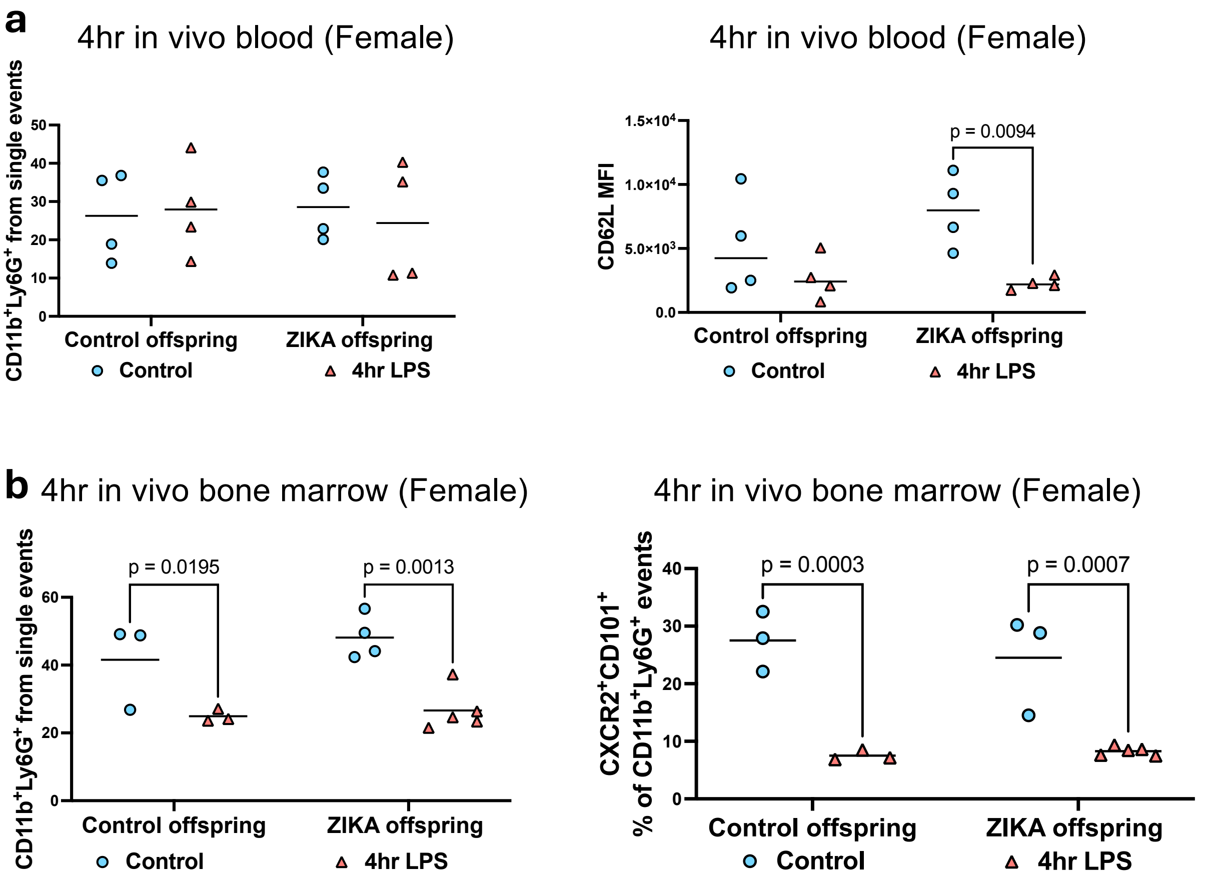

Supplemental Figure 4. Effect of maternal Zika infection on female offspring neutrophil response to LPS.

Female offspring from control and Zika-exposed groups were treated with lipopolysaccharide (LPS) (0.8 mg/kg) intraperitoneally at PND35. (a) Percentage of CD11b$^+$Ly6G$^+$ neutrophil populations and CD62L mean fluorescence intensity (MFI) of neutrophils in the blood at 4 hours post-LPS treatment. *n* = 4 mice/group. (b) Percentage of CD11b$^+$Ly6G$^+$ neutrophil populations and mature neutrophil subsets CXCR2$^+$CD101$^+$ from CD11b$^+$Ly6G$^+$ in the bone marrow at 4 hours post-LPS treatment. *n* = 3 mice for control untreated and LPS group, *n* = 3 or 4 mice for ZIKV untreated and 5 mice for ZIKV LPS group. Data are shown as the mean ± SD. Two-way ANOVA with Šídák's multiple comparisons was used.

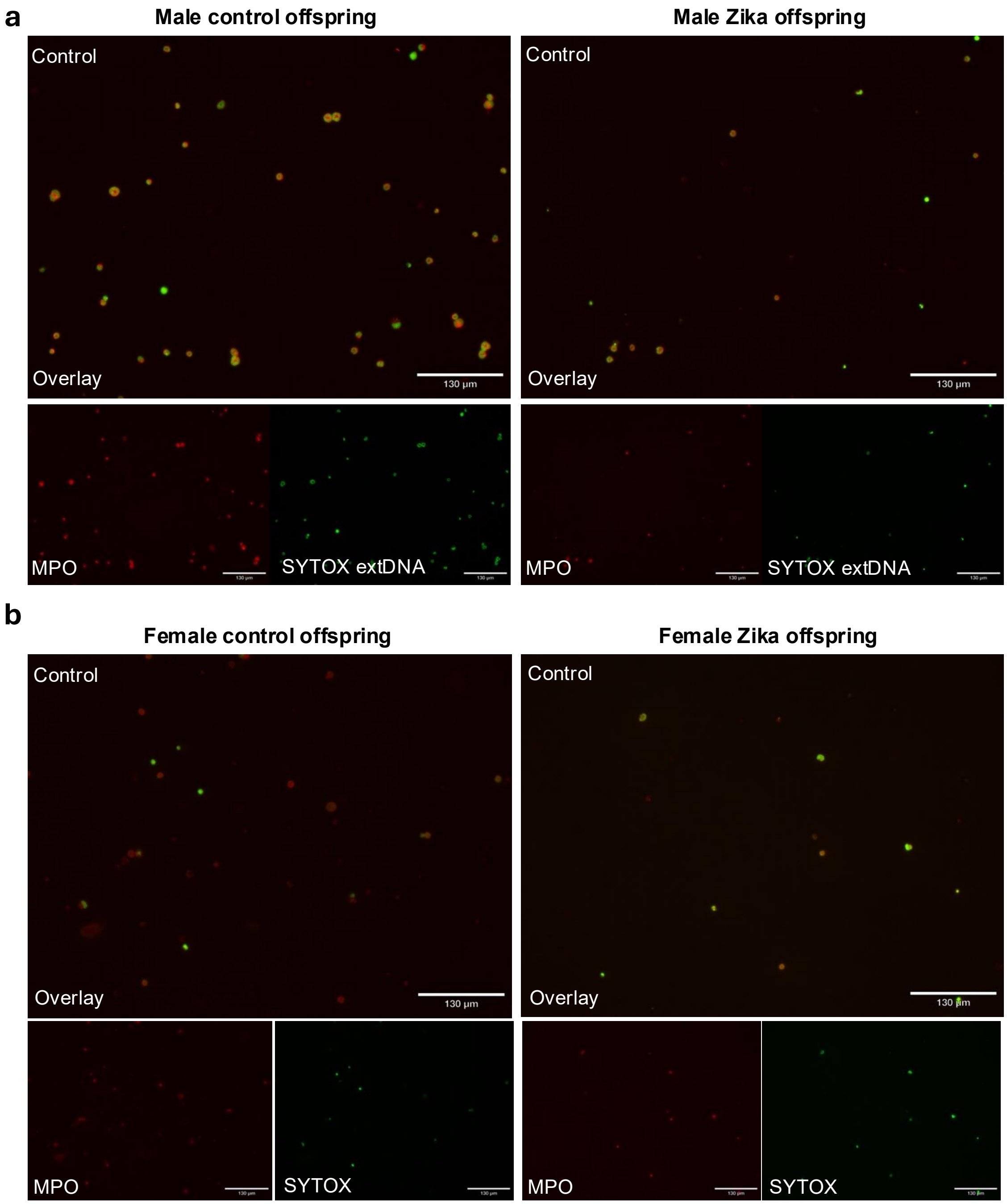

Supplemental Figure 5. Representative image of controls in NETs immunofluorescence experiment. Red: MPO; green: SYTOX, indicating extracellular DNA. Co-localization of SYTOX Green and MPO confirmed the presence of NETs. Scale bar = 130 µm. *n* = 1 isolation from 3 mice, and cells were plated in triplicate.

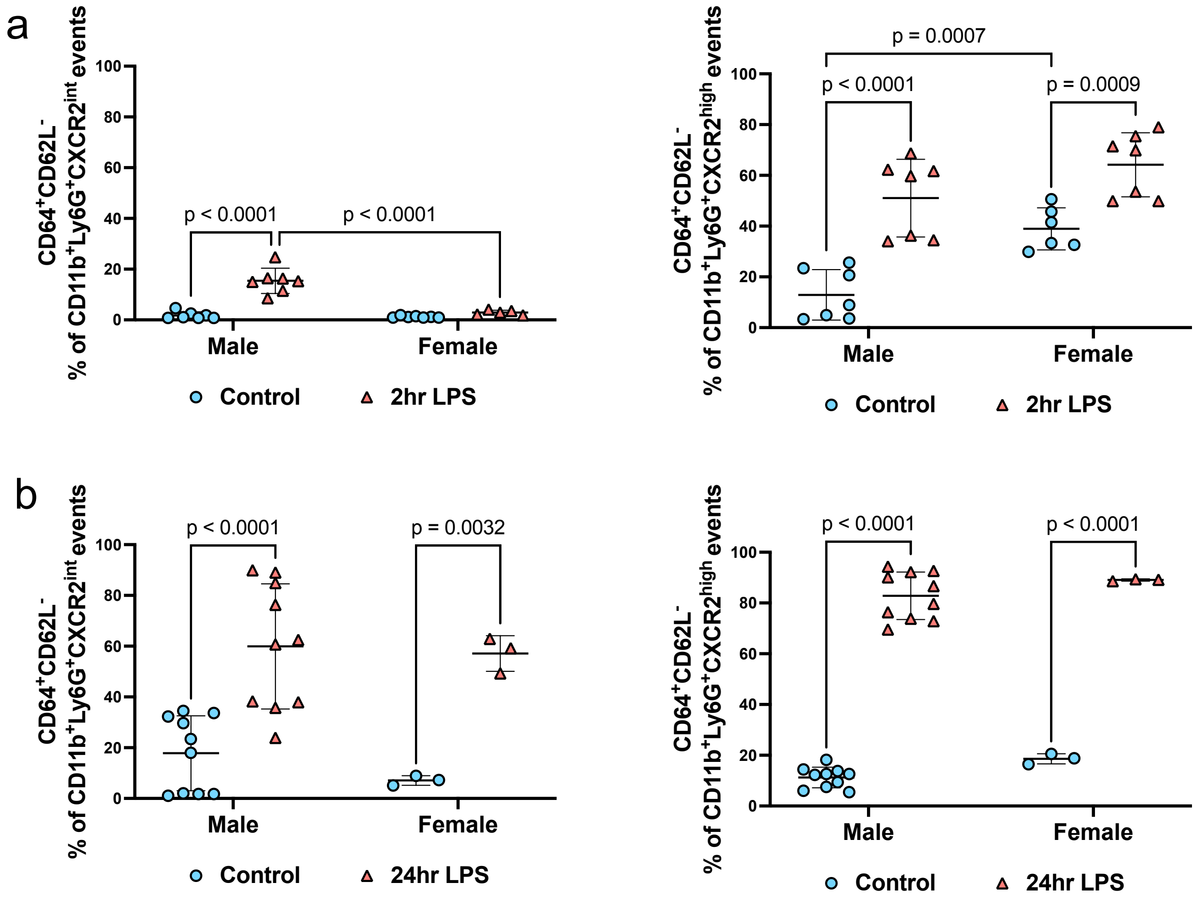

Supplemental Figure 6. Sex-dimorphic response to LPS in female and male bone marrow neutrophils. (a) Percentage of activated neutrophils ($CD64^+CD62L^-$) within $CXCR2^{int}$ and $CXCR2^{high}$ populations after 2 hours of LPS treatment, compared between male and female neutrophils. *n* = 3 isolations/group. (b) Percentage of activated neutrophils ($CD64^+CD62L^-$) within $CXCR2^{int}$ and $CXCR2^{high}$ populations after 24 hours of LPS treatment, compared between male and female neutrophils. *n* = 4 isolations/group. and each isolation pooled neutrophils from 2–3 mice and was plated in duplicate or triplicate. Data are shown as the mean ± SD. Two-way ANOVA with Šídák's multiple comparisons was used.

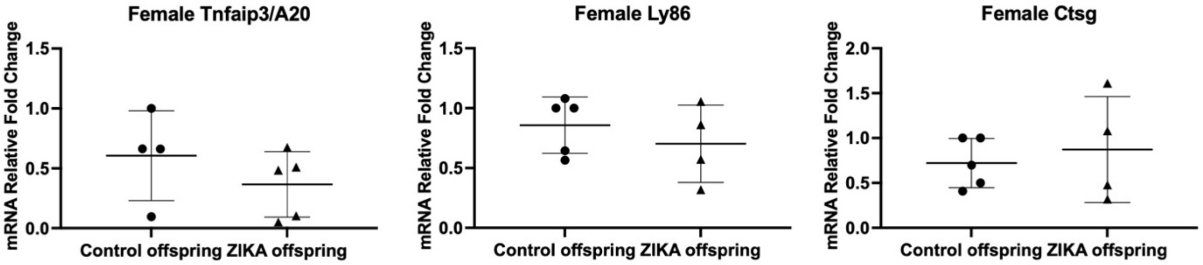

Supplemental Figure 7. Validation of Tnfaip3/A20, Ly86 and Ctsg mRNA expression in female mouse neutrophils via qRT-PCR. Data are shown as the mean ± SD. Two-sided Student's t-test was used. *n* = 4 control, 5 ZIKV for Tnfaip3/A20; *n* = 5 control, 4 ZIKV for Ly86 and Ctsg. n denotes independent isolations pooled from 2–3 mice and run in duplicate for qRT-PCR.

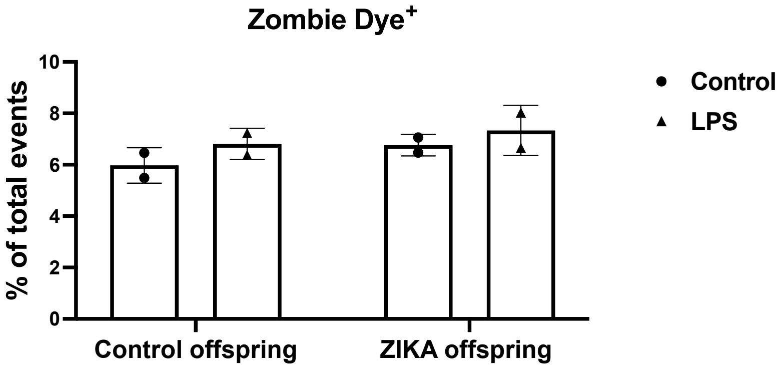

Supplemental Figure 8. Viability of isolated neutrophils after 24 hours of LPS treatment. Zombie Dye+ cells indicated the dead neutrophils after 24 hours of LPS treatment. *n* = 2, neutrophils were isolated from 2 mice per group across 2 independent experiments. Data are presented as mean ± SD.

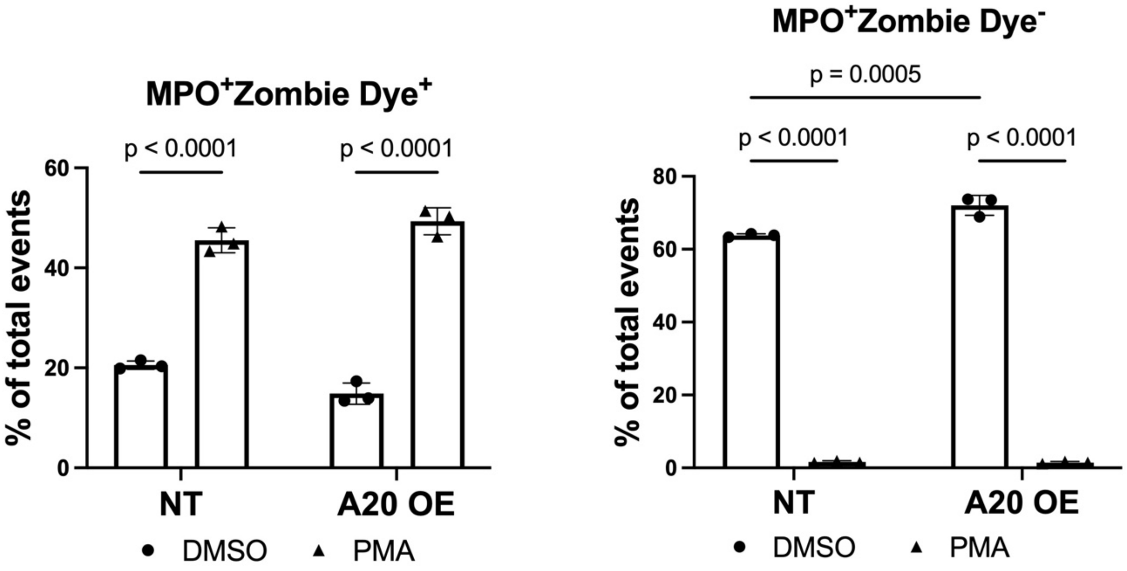

Supplemental Figure 9. Viability of isolated neutrophils after 24 hours of PMA treatment. Percentage of non-viable neutrophils (MPO^+Zombie Dye^+) and viable neutrophils (MPO^+Zombie Dye^-) after 24 hours of PMA treatment. n = 3, one isolation from 2 mice and plated in triplicates. Data are presented as mean ± SD. Two-way ANOVA with Šídák's multiple comparisons was used. NT: no treatment; OE: overexpression.

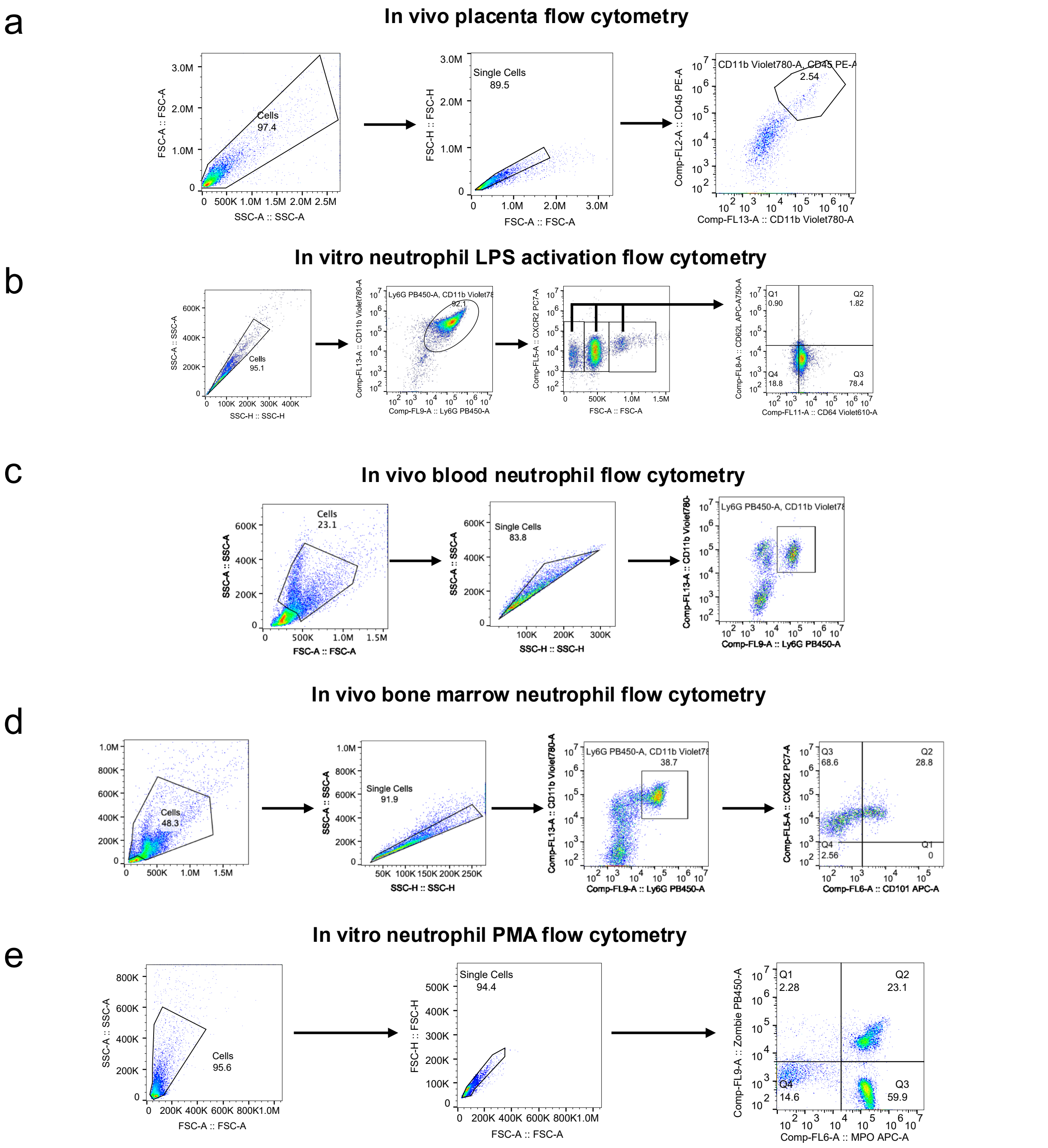

Supplemental Figure 10. Gating strategy for flow cytometry analysis.

Panel (a) corresponds to gating strategy applied in Figure 2. Panels (b), (c), (d), and (e) correspond to gating strategies used in Figures 7c–f, 4c, 4d, and 8f, respectively.

Gene	Forward (5'-3')	Reverse (5'-3')
Mpo	CGTGTCAAGTGGCTGTGCCTAT	AACCAGCGTACAAAGGCACGGT
Elane	CAGGAACTTCGTCATGTCAGCAG	AGCCATTCTCGAAGATCCGCTG
Ly86	GCCTTTCCATTGACCAGTGTTCC	GCCTTTTGCCATCAGAGTTATGTC
Ctsg	AGTCCAGAAGGGCTGAGTGCTT	GCACTGTGATGAGTTGCTGGGT
Tnfaip3 (A20)	AGCAAGTGCAGGAAAGCTGGCT	GCTTTCGCAGAGGCAGTAACAG
DDX3Y	TGTTAGTTGCCACACCAGGACG	TGGTGGCATTGTGTCCTGCTCA
PPIA	CATACAGGTCCTGGCATCTTGTC	AGACCACATGCTTGCCATCCAG

Supplemental Table 3. Primer sequences for qRT-PCR.
