# Supplementary material for: Prenatal exposure to Zika virus shapes offspring neutrophil function in a sex-specific manner
Source: Nat Commun. 2025 Oct 3;16:8839. doi: 10.1038/s41467-025-63941-x (PMC12494944; doi:10.1038/s41467-025-63941-x)
Supplement: Supplementary file 2 — Reporting summary [file 41467_2025_63941_MOESM2_ESM.pdf]

nature portfolio

Corresponding author(s): Jiahui Ding

Last updated by author(s): Aug 12, 2025

Reporting Summary

Nature Portfolio wishes to improve the reproducibility of the work that we publish. This form provides structure for consistency and transparency in reporting. For further information on Nature Portfolio policies, see our Editorial Policies and the Editorial Policy Checklist.

Statistics

For all statistical analyses, confirm that the following items are present in the figure legend, table legend, main text, or Methods section.

n/a	Confirmed	
☐	☒	The exact sample size (*n*) for each experimental group/condition, given as a discrete number and unit of measurement
☐	☒	A statement on whether measurements were taken from distinct samples or whether the same sample was measured repeatedly
☐	☒	The statistical test(s) used AND whether they are one- or two-sided<br>*Only common tests should be described solely by name; describe more complex techniques in the Methods section.*
☐	☒	A description of all covariates tested
☐	☒	A description of any assumptions or corrections, such as tests of normality and adjustment for multiple comparisons
☐	☒	A full description of the statistical parameters including central tendency (e.g. means) or other basic estimates (e.g. regression coefficient) AND variation (e.g. standard deviation) or associated estimates of uncertainty (e.g. confidence intervals)
☐	☒	For null hypothesis testing, the test statistic (e.g. *F*, *t*, *r*) with confidence intervals, effect sizes, degrees of freedom and *P* value noted<br>*Give P values as exact values whenever suitable.*
☒	☐	For Bayesian analysis, information on the choice of priors and Markov chain Monte Carlo settings
☐	☒	For hierarchical and complex designs, identification of the appropriate level for tests and full reporting of outcomes
☒	☐	Estimates of effect sizes (e.g. Cohen's *d*, Pearson's *r*), indicating how they were calculated

Our web collection on statistics for biologists contains articles on many of the points above.

Software and code

Policy information about availability of computer code

Data collection	LUMINEX xPONENT software (v4.3),CytExpert (RRID : SCR_017217) acquisition software (v2.6)
Data analysis	GraphPad Prism (v10.4.0), Image J software (1.49v), FlowJo (RRID : SCR_008520; v10.10.0), iPathwayGuide software (v18.1), Trimmomatic (v0.36.88), Hisat2 (v2.2.1) , Subread package (v1.5.2.90), DESeq2 (v1.36.0), R (v4.4.0), Rstudio (2024.09.0+375).ComplexHeatmap (v2.22.0), PCAtools (v2.20.0), ggplot2 (v3.4.0).

For manuscripts utilizing custom algorithms or software that are central to the research but not yet described in published literature, software must be made available to editors and reviewers. We strongly encourage code deposition in a community repository (e.g. GitHub). See the Nature Portfolio guidelines for submitting code & software for further information.

Data

Policy information about availability of data

All manuscripts must include a data availability statement. This statement should provide the following information, where applicable:

- Accession codes, unique identifiers, or web links for publicly available datasets
- A description of any restrictions on data availability
- For clinical datasets or third party data, please ensure that the statement adheres to our policy

The raw and processed RNA sequence data form our mouse placentas and neutrophil experiments are available in NCBI's Gene Expression Omnibus under the accession number GSE292966 (https://www.ncbi.nlm.nih.gov/geo/query/acc.cgi?acc=GSE292966). Source Data are provided with this paper and deposited in Figshare at https://doi.org/10.6084/m9.figshare.29538431.

Research involving human participants, their data, or biological material

Policy information about studies with human participants or human data. See also policy information about sex, gender (identity/presentation), and sexual orientation and race, ethnicity and racism.

Reporting on sex and gender	*Use the terms sex (biological attribute) and gender (shaped by social and cultural circumstances) carefully in order to avoid confusing both terms. Indicate if findings apply to only one sex or gender; describe whether sex and gender were considered in study design; whether sex and/or gender was determined based on self-reporting or assigned and methods used.*<br>*Provide in the source data disaggregated sex and gender data, where this information has been collected, and if consent has been obtained for sharing of individual-level data; provide overall numbers in this Reporting Summary. Please state if this information has not been collected.*<br>*Report sex- and gender-based analyses where performed, justify reasons for lack of sex- and gender-based analysis.*
Reporting on race, ethnicity, or other socially relevant groupings	*Please specify the socially constructed or socially relevant categorization variable(s) used in your manuscript and explain why they were used. Please note that such variables should not be used as proxies for other socially constructed/relevant variables (for example, race or ethnicity should not be used as a proxy for socioeconomic status).*<br>*Provide clear definitions of the relevant terms used, how they were provided (by the participants/respondents, the researchers, or third parties), and the method(s) used to classify people into the different categories (e.g. self-report, census or administrative data, social media data, etc.)*<br>*Please provide details about how you controlled for confounding variables in your analyses.*
Population characteristics	*Describe the covariate-relevant population characteristics of the human research participants (e.g. age, genotypic information, past and current diagnosis and treatment categories). If you filled out the behavioural & social sciences study design questions and have nothing to add here, write "See above."*
Recruitment	*Describe how participants were recruited. Outline any potential self-selection bias or other biases that may be present and how these are likely to impact results.*
Ethics oversight	*Identify the organization(s) that approved the study protocol.*

Note that full information on the approval of the study protocol must also be provided in the manuscript.

Field-specific reporting

Please select the one below that is the best fit for your research. If you are not sure, read the appropriate sections before making your selection.

☒ Life sciences ☐ Behavioural & social sciences ☐ Ecological, evolutionary & environmental sciences

For a reference copy of the document with all sections, see nature.com/documents/nr-reporting-summary-flat.pdf

Life sciences study design

All studies must disclose on these points even when the disclosure is negative.

Sample size	Sample sizes were determined using power analysis (G*Power 3.1) based on preliminary experimental data, aiming for adequate statistical power to detect significant differences between groups.
Data exclusions	Outliers (data points > 2 standard deviations from the mean) were excluded. This exclusion criterion was pre-established to reduce the influence of extreme values on statistical outcomes.
Replication	All key experiments were performed with at least three biological replicates. Technical replicates were included as appropriate to ensure measurement consistency.

Randomization	For all experiments, mice were randomly assigned to different groups to minimize selection bias.
Blinding	Data collection (e.g., sample processing, imaging, and assay testing) was performed in a blinded manner. Investigators were blinded to group allocation during data collection by coding samples.

Reporting for specific materials, systems and methods

We require information from authors about some types of materials, experimental systems and methods used in many studies. Here, indicate whether each material, system or method listed is relevant to your study. If you are not sure if a list item applies to your research, read the appropriate section before selecting a response.

Materials & experimental systems

n/a	Involved in the study
☐	☒ Antibodies
☒	☐ Eukaryotic cell lines
☒	☐ Palaeontology and archaeology
☐	☒ Animals and other organisms
☒	☐ Clinical data
☒	☐ Dual use research of concern
☒	☐ Plants

Methods

n/a	Involved in the study
☒	☐ ChIP-seq
☐	☒ Flow cytometry
☒	☐ MRI-based neuroimaging

Antibodies

Antibodies used	Flow antibody from BioLegend: CD11b (M1/70, #101243), Ly6G (1A8, #127612), CD45 (30-F11, #103114), CXCR2 (SA045E1, #149618), CD62L (MEL-14, #104450), CD64 (X54-5/7.1, #139323). MPO (8F4/MPO, #570233, BD Biosciences), CD101 (307707, #564473, BD Biosciences), . Anti-myeloperoxidase (MPO) antibody (R&D, Cat# AF3667).A20 (Cell Signaling Technology, catalog no. 5630); 1:10,000 anti-GAPDH (Sigma, catalog no. G8795); 1:10,000 anti-beta-Actin (Cell Signaling Technology, catalog no. 4967); and 1:10,000 peroxidase- conjugated anti-rabbit IgG (Cell Signaling Technology, catalog no. 7074); and 1:10,000 peroxidase- conjugated anti-mouse IgG (Cell Signaling Technology, catalog no. 7076) .
Validation	Antibodies used in Western blotting, immunofluorescence, and flow cytometry were validated for specificity and sensitivity by following manufacturer-provided validation data and confirmed by detecting target proteins at expected molecular weights (Western blot) or expected subcellular localization patterns (immunofluorescence). For flow cytometry, antibody performance was assessed using appropriate gating strategies, including positive controls to ensure signal specificity. All antibodies used in this study have been previously validated in peer-reviewed publications or by the manufacturer for the relevant applications.<br>Validation:<br>CD11b: Sanmarco LM, et al. 2022. Nature. 611:801. Ly6G:Hou X, et al. 2020. Cell Reports. 28(1):172-189.e7. CD45: Tran NT, et al. 2019. Cell Rep. 28:3510. CXCR2:Natsuaki Y, et al. 2014. Nat. Immunol. 15:1064. CD62L: Cardinez C, et al. 2024. Nat Commun. 2345:15. CD64: Bhattacharjee A, et al. 2021. Immunity. 54(8):1745-1757.e7. MPO from BD for fLow cytometry: Klebanoff SJ. Myeloperoxidase: friend and foe. J Leukoc Biol. 2005; 77(5):598-625. CD101: Yang, F., Suo, M., et al. Staphylococcus aureus α-toxin impairs early neutrophil localization via electrogenic disruption of store-operated calcium entry. Cell Reports. 11.28.2023. MPO from R&D for IF: https://www.rndsystems.com/products/human-mouse-myeloperoxidase-mpo-antibody_af3667#product-citations. A20:Lee, E.G. et al. (2000) Science 289, 2350-4. GAPDH: https://www.sigmaaldrich.com/US/en/product/sigma/g8795?srsltid=AfmBOorqvPxhbGP1_WEEDO4huCEAqz-4dn32lUL3Q7efCPrIPufl_Vi_. Beta-actin: Kayalar, C. et al. (1996) Proc Natl Acad Sci U S A 93, 2234-8.

Animals and other research organisms

Policy information about studies involving animals; ARRIVE guidelines recommended for reporting animal research, and Sex and Gender in Research

Laboratory animals	C57BL/6 mice, for pregnancy experiment (8-10 weeks old, female and male); for offspring experiment (5-6 weeks old, female and male).
Wild animals	*Provide details on animals observed in or captured in the field; report species and age where possible. Describe how animals were caught and transported and what happened to captive animals after the study (if killed, explain why and describe method; if released, say where and when) OR state that the study did not involve wild animals.*
Reporting on sex	Yes, both female and male mice were included.
Field-collected samples	*For laboratory work with field-collected samples, describe all relevant parameters such as housing, maintenance, temperature, photoperiod and end-of-experiment protocol OR state that the study did not involve samples collected from the field.*

Ethics oversight	All procedures were performed in accordance with National Institutes of Health guidelines (NIH Publication No. 85–23, revised 1996) and approved by the Institutional Animal Care and Use Committee (IACUC) at Wayne State University under protocol number 25-02-7578.

Note that full information on the approval of the study protocol must also be provided in the manuscript.

Plants

Seed stocks	*Report on the source of all seed stocks or other plant material used. If applicable, state the seed stock centre and catalogue number. If plant specimens were collected from the field, describe the collection location, date and sampling procedures.*
Novel plant genotypes	*Describe the methods by which all novel plant genotypes were produced. This includes those generated by transgenic approaches, gene editing, chemical/radiation-based mutagenesis and hybridization. For transgenic lines, describe the transformation method, the number of independent lines analyzed and the generation upon which experiments were performed. For gene-edited lines, describe the editor used, the endogenous sequence targeted for editing, the targeting guide RNA sequence (if applicable) and how the editor was applied.*
Authentication	*Describe any authentication procedures for each seed stock used or novel genotype generated. Describe any experiments used to assess the effect of a mutation and, where applicable, how potential secondary effects (e.g. second site T-DNA insertions, mosiacism, off-target gene editing) were examined.*

Flow Cytometry

Plots

Confirm that:

- [x] The axis labels state the marker and fluorochrome used (e.g. CD4-FITC).
- [x] The axis scales are clearly visible. Include numbers along axes only for bottom left plot of group (a 'group' is an analysis of identical markers).
- [x] All plots are contour plots with outliers or pseudocolor plots.
- [x] A numerical value for number of cells or percentage (with statistics) is provided.

Methodology

Sample preparation	Placenta: Placental tissues were mechanically minced and enzymatically digested using collagenase type I (1 mg/mL) at 37°C for 60 minutes with gentle agitation to obtain a single-cell suspension. The digest was filtered through a 70 µm cell strainer to remove debris before staining.<br>Bone marrow neutrophils: Bone marrow cells were harvested from mouse femurs and tibias and neutrophils were isolated using the Miltenyi Biotec Neutrophil Isolation Kit according to the manufacturer's instructions. Purity and viability were confirmed by flow cytometry prior to analysis.<br>Blood: Peripheral blood was collected and red blood cells were lysed using RBC lysis buffer before washing and staining for flow cytometry.
Instrument	Data were acquired using CytoFLEX analyzer (RRID : SCR_019627).
Software	Data were collected using CytExpert (RRID : SCR_017217) acquisition software (Beckman Coulter, Brea, CA). Data were analyzed using FlowJo (RRID : SCR_008520; Becton, Dickinson and Company, Ashland, OR).
Cell population abundance	The purity of the isolated neutrophils was assessed by flow cytometry using CD11b and Ly6G staining, with purity exceeding 95%.
Gating strategy	The initial gate to identify the target cell population was set on forward scatter area (FSC-A) versus side scatter area (SSC-A) plots to exclude debris and select cells based on size and granularity. Doublets were excluded by gating on FSC-A versus FSC-H or SSC-A versus SSC-H plots, depending on the experiment.<br>For defining positive versus negative populations, single-stained positive and negative control samples were used to set fluorescence thresholds. Gates were established conservatively based on these controls to distinguish clearly between stained (positive) and unstained (negative) populations. The same gating strategy was consistently applied to all samples within each experiment.

- [x] Tick this box to confirm that a figure exemplifying the gating strategy is provided in the Supplementary Information.
